# Supplementary material for: Correction: Energy and Cost Associated with Ventilating Office Buildings in a Tropical Climate
Source: PLoS One. 2015 May 1;10(5):e0127930. doi: 10.1371/journal.pone.0127930 (PMC4416807; doi:10.1371/journal.pone.0127930)
Supplement: S1 File — (PDF) [file pone.0127930.s001.pdf]

RESEARCH ARTICLE

Energy and Cost Associated with Ventilating Office Buildings in a Tropical Climate

Donghyun Rim[1¤*], **Stefano Schiavon**[2], **William W. Nazaroff**[1]

1 Department of Civil and Environmental Engineering, University of California, Berkeley, California, United States of America, **2** Department of Architecture, University of California, Berkeley, California, United States of America

¤ Current address: Department of Architectural Engineering, Pennsylvania State University, University Park, Pennsylvania, United States of America
* drim@psu.edu

CrossMark
click for updates

OPEN ACCESS

Citation: Rim D, Schiavon S, Nazaroff WW (2015) Energy and Cost Associated with Ventilating Office Buildings in a Tropical Climate. PLoS ONE 10(3): e0122310. doi:10.1371/journal.pone.0122310

Academic Editor: Maosheng Yao, Peking University, CHINA

Received: September 12, 2014

Accepted: February 19, 2015

Published: March 30, 2015

Copyright: © 2015 Rim et al. This is an open access article distributed under the terms of the Creative Commons Attribution License, which permits unrestricted use, distribution, and reproduction in any medium, provided the original author and source are credited.

Data Availability Statement: All relevant data are within the paper.

Funding: This research is funded by the Republic of Singapore's National Research Foundation through a grant to the Berkeley Education Alliance for Research in Singapore (BEARS) for the Singapore-Berkeley Building Efficiency and Sustainability in the Tropics (SinBerBEST) Program. BEARS has been established by the University of California, Berkeley as a center for intellectual excellence in research and education in Singapore.

Competing Interests: The authors have declared that no competing interests exist.

Abstract

Providing sufficient amounts of outdoor air to occupants is a critical building function for supporting occupant health, well-being and productivity. In tropical climates, high ventilation rates require substantial amounts of energy to cool and dehumidify supply air. This study evaluates the energy consumption and associated cost for thermally conditioning outdoor air provided for building ventilation in tropical climates, considering Singapore as an example locale. We investigated the influence on energy consumption and cost of the following factors: outdoor air temperature and humidity, ventilation rate (L/s per person), indoor air temperature and humidity, air conditioning system coefficient of performance (COP), and cost of electricity. Results show that dehumidification of outdoor air accounts for more than 80% of the energy needed for building ventilation in Singapore's tropical climate. Improved system performance and/or a small increase in the indoor temperature set point would permit relatively large ventilation rates (such as 25 L/s per person) at modest or no cost increment. Overall, even in a thermally demanding tropical climate, the energy cost associated with increasing ventilation rate up to 25 L/s per person is less than 1% of the wages of an office worker in an advanced economy like Singapore's. This result implies that the benefits of increasing outdoor air ventilation rate up to 25 L/s per person — which is suggested to provide for productivity increases, lower sick building syndrome symptom prevalence, and reduced sick leave — can be much larger than the incremental cost of ventilation.

Introduction

Ventilation is important for the health, well-being and productivity of building occupants [1–3]. Outdoor ventilation air is supplied to dilute contaminants of indoor origin and to replace the air exhausted from a building. If conditions in outdoor air are not suitable for human thermal comfort needs, then energy must be expended to alter the temperature and/or humidity of the ventilation air. In a tropical climate, energy requirements for thermal conditioning of ventilation air are potentially high. However, ventilation rates that are too low can cause increased

risks of adverse health effects for building occupants or otherwise contributed to degraded conditions for occupant well-being.

Associations of insufficient building ventilation with adverse health effects have been well documented [1–8]. Adverse health outcomes associated with low ventilation rates include increased prevalence of sick building syndrome (SBS) symptoms [2,8], respiratory disease [9], allergic symptoms [10], and sensory irritation [5, 11]. Furthermore, insufficient ventilation of occupied spaces can increase the transmission risk for certain infectious diseases [6]. These outcomes adversely affect occupant health and productivity, contributing to increased health care cost, productivity loss, and absenteeism.

Detailed consideration of how outdoor air is distributed within buildings is an important aspect of ventilation system performance [12–14]. However, irrespective of the efficiency of delivery, a fundamental consideration for achieving good indoor air quality pertains to the sufficiency of ventilation. Based on an extensive multidisciplinary literature review of the ventilation and health, Sundell et al. [1] reported that higher than usual ventilation rates in offices, up to about 25 L/s per person, were associated with reduced prevalence of SBS symptoms.

In Singapore's tropical climate, a government standard [15, 16] specifies recommended minimum ventilation rates (e.g., 5.5 L/s per person for offices). In the United States, requirements for ventilation of non-residential buildings are based on ASHRAE Standard 62.1–2013 [17]. This standard specifies a minimum ventilation rate of 10 L/s per person for offices. For different countries, the standards and guidelines specifying minimum ventilation rates in buildings vary based on building type, climate, principal activity, indoor pollution sources, and density of occupants [17–20]. However, typically these are minimum standards that do not necessarily reflect levels of ventilation that might be desired to promote occupant health, productivity, and well-being. Furthermore, several studies have reported that a significant fraction of buildings fail even to meet the minimum ventilation rates specified in current standards and guidelines [21–23].

In developed countries, buildings account for a significant portion of primary energy consumption [23–27]. For example, buildings are estimated to be responsible for 72% and 50% of end-use electricity consumption in the US and Singapore, respectively [28, 29]. The outdoor ventilation rates for large commercial office buildings are generally controlled by a heating, ventilating and air-conditioning (HVAC) system, which can be a key contributor (40–50%) to overall building energy consumption [25, 26, 29]. Conditioning and supplying outdoor air in a tropical climate may have significant impact on building energy consumption, given the relatively high energy required for cooling and dehumidifying outdoor air during the whole year. Furthermore, in tropical climate zones, owing to rapid population growth and urbanization [25, 30] along with the influence of climate change [31, 32], building energy use is expected to have growing influence on both local and global energy consumption rates [33, 34]. Considering these trends, developing energy efficient and sustainable buildings for tropical climates is an urgent need. Pursuing the goal to limit energy use might result in a systematic reduction of the ventilation rates of buildings. Reducing ventilation rates is clearly beneficial for saving energy and for lowering associated costs. However, inadequate ventilation rates can compromise indoor environmental quality and adversely affect building occupants [35]. An important issue is how much outdoor air should be supplied to a building to promote occupant health, well-being, productivity, while still limiting energy consumption and maintaining affordable cost.

In light of the challenge of balancing energy efficiency goals with health and well-being concerns for building occupants, the objective of this study is to evaluate the energy consumption and financial costs associated with providing ventilation for office buildings in a tropical climate. Considering outdoor conditions for Singapore as a particular example, this study

considers the fundamental aspects of energy consumption and cost associated with particular building ventilation rates.

Methods

This study calculates energy consumption rates (sensible and latent energy, in kWh/y per person) associated with a range of ventilation rates. The energy consumption for outdoor air ventilation was calculated as a function of the following variables: ventilation rate (L/s per person), indoor dry-bulb set-point temperature and relative humidity (RH), and air conditioning system coefficient of performance (COP). The metric of ventilation rate per person is widely used as a design criterion in the HVAC industry for performance-based building standards and codes.

The calculation is carried out for the specific climatic conditions in Singapore. The evaluation is performed for a whole year assuming constant air flow rate during typical operating hours (M-F 7 am to 7 pm) for office buildings in Singapore [36]. In this section, the outdoor weather and building conditions in the analytical model are presented, followed by a description of the calculation procedure. Subsequently, the interpretations of building ventilation energy consumption along with assumptions for the analysis are discussed.

Outdoor weather data and input parameters

Annual hourly weather data for Singapore were retrieved from the Southwest Pacific WMO Region 5 of the IWEC (International Weather for Energy Calculations). The weather data set was derived from up to 18 years of hourly weather data originally archived at the US National Climatic Data Center [37]. Fig 1 shows annual patterns of temperature, humidity mass ratio, and specific enthalpy for outdoor air in Singapore. The hourly outdoor temperature ranges from 21.0°C to 33.8°C for the year with a mean of 28.7°C. The humidity mass ratio varies from 0.012 to 0.024 kg of water vapor per kg of dry air with a mean of 0.019 kg_w/kg_{da}. The resulting specific enthalpy of outdoor air (as computed from the equation in Table 1) ranges between 61 and 91 kJ/kg_{da} with a mean of 77 kJ/kg_{da}. Comparing outdoor conditions for the building operating hours (7 am to 7 pm) to the entire 24-h period, the outdoor dry-bulb temperature is slightly higher (~1.3°C) for the building operating hours, while the differences in humidity ratio and enthalpy are small (< 2%).

Calculating ventilation energy use and associated cost

Using the outdoor data, several psychrometric parameters (Table 1) were calculated to determine the amount of energy necessary for conditioning outdoor ventilation air to the indoor set-point temperature and relative humidity. Those parameters include the specific enthalpy of moist air and the sensible energy and latent energy needed to condition outdoor ventilation air. The specific enthalpy of moist air (h, kJ/kg_{da}) was calculated based on the specific heat of dry air and water vapor along with the enthalpy change attributable to water vaporization. The cooling process is analyzed separately for sensible and latent energy. Hourly sensible energy was estimated considering specific heat of moist air and the indoor-outdoor temperature change. Hourly latent energy was calculated based on the difference between the indoor and outdoor humidity ratios and the enthalpy change due to water vaporization. The calculations considered ventilation rates ranging from 10 L/s per person to 40 L/s per person. The lower end of this range represents the minimum ventilation requirements for ASHRAE 62.1–2013 for an office with an occupant density of 25 m^2 per person [17], whereas the higher ventilation rates (> 25 L/s per person) correspond to levels that have been found to be associated with lower building-related symptoms and improved worker productivity [1–2, 6–8, 39].

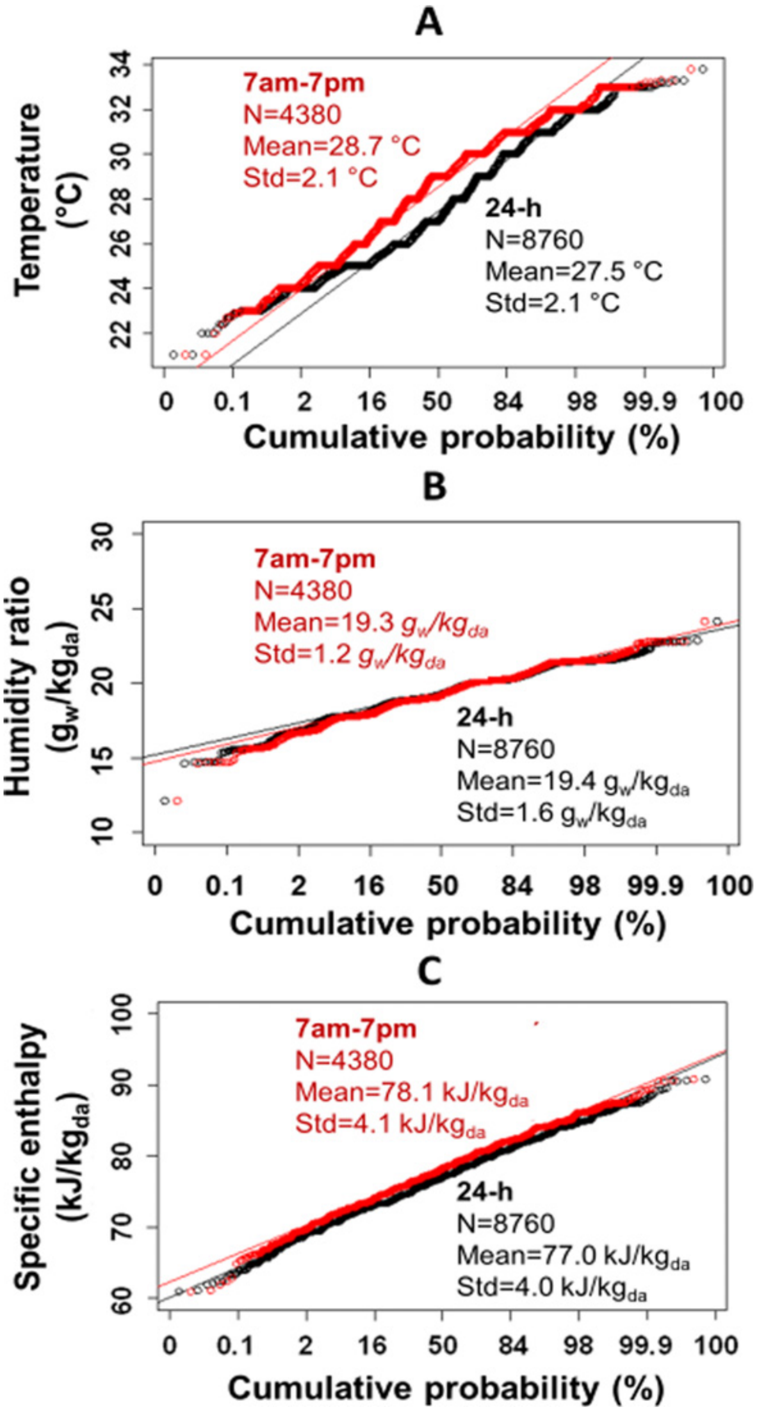

Fig 1. Annual outdoor weather conditions for Singapore. (A) dry-bulb air temperature (°C). (B) humidity ratio (in units of g of H_2O vapor per kg of dry air). (C) specific enthalpy (kJ per kg of dry air).

doi:10.1371/journal.pone.0122310.g001

We considered these ranges of indoor set-point conditions: temperature = 22–28°C and RH = 20–80%. In principle, significant energy savings can be realized in tropical climates by increasing the set-point temperature and by using enhanced air movement to provide augmented cooling [41–45]. On this basis, and to consider the potential for pursuing energy efficiency

Table 1. Psychrometric parameters estimated in the ventilation energy analysis.

Parameter	Equation
Saturation vapor pressure (P_{ws}, Pa) [40]	$P_{ws} = 1000 \times exp\left(16.7 - \frac{4060}{T_a - 37}\right)$
	where T_a = absolute temperature, K (= T + 273.15); T = dry-bulb temperature, °C
Water vapor pressure (P_w, Pa)	$P_w = P_{ws} \cdot \emptyset$
	where P_{ws} = saturation vapor pressure, Pa; $\emptyset$ = relative humidity (RH)
Density of dry air (ρ_{da}, kg_{da}/m^3)	$\rho_{da} = \frac{28.97\ (P - P_w)}{RT_a}$
	where R = universal gas constant, 8.314 J/(mol·K)
Humidity ratio (W, kg_w/kg_{da})	$W = 0.622 \frac{P_w}{P - P_w}$
	where P = atmospheric pressure (101325 Pa for sea level); P_w = water vapor pressure (Pa)
Specific enthalpy of moist air (h, kJ/kg_{da}, referenced to 0°C) [38]	$h = [C_{pa} \cdot T + W \cdot (h_{fg} + C_{pw} \cdot T)]$
	where W = humidity ratio, kg_w/kg_{da}; C_{pa} = specific heat of dry air, 1.006 kJ/(kg·K); C_{pw} = specific heat of water vapor, 1.86 kJ/(kg·K); h_{fg} = enthalpy change due to water vaporization, 2500 kJ/kg water (approximate heat content of 50% RH vapor at 24°C less the heat content of water at 10°C).
Sensible energy ($E_{sensible}$, kJ) [38]	$E_{sensible} = \rho_{da} \cdot Q \cdot (C_{pa} + W \cdot C_{pw}) \cdot \Delta T \cdot \Delta t$
	where ρ_{da} = density of dry air (kg/m^3); Q = airflow rate, m^3/s; C_{pa} = specific heat of dry air, 1.006 kJ/(kg·K); C_{pw} = specific heat of water vapor, 1.86 kJ/(kg·K); ΔT = indoor-outdoor temperature difference (K); Δt = duration of analysis period (s)
Latent energy (E_{latent}, kJ) [38]	$E_{latent} = \rho_{da} \cdot Q \cdot \Delta W \cdot h_{fg} \cdot \Delta t$
	where ΔW = indoor-outdoor humidity ratio difference, kg_w/kg_{da}

doi:10.1371/journal.pone.0122310.t001

goals with higher than normal set points, a wide range of indoor temperature and RH values were considered.

The psychrometric parameters in Table 1 were calculated on an hourly basis using both indoor set points (fixed) and outdoor conditions (time varying). Based on the indoor-outdoor enthalpy difference of air along with the specified ventilation rate (L/s per person), annual ventilation-specific energy use was calculated in units of kWh/y per person. Considering the variation in the air-conditioning system efficiency, coefficient-of-performance (COP) values were considered across the range of 2 to 6. Modern system equipment can attain a COP higher than 4, while the value for inefficient systems is typically 2 to 3. The operating cost for treating ventilation air also depends on the cost of electricity; we assumed values in the range US$0.20/kWh—$0.40/kWh. The COP and electricity prices are consistent with current and expected future values for Singapore [46, 47].

Assumptions and interpretation

The present study evaluates the energy required for thermally conditioning outdoor ventilation air. Ventilation-specific energy consumption needed to bring the outdoor air to the set-point temperature and humidity conditions was estimated so as to obtain results that are independent of specific building features or mechanical system characteristics. The calculation of energy required for ventilation is made based on a constant indoor set-point condition and outdoor ventilation flow rate during all hours of building operation. In Singapore's tropical climate, outdoor conditions are consistently warm and humid throughout the year. However, for climates that vary seasonally, buildings would likely operate with different set-point temperatures in

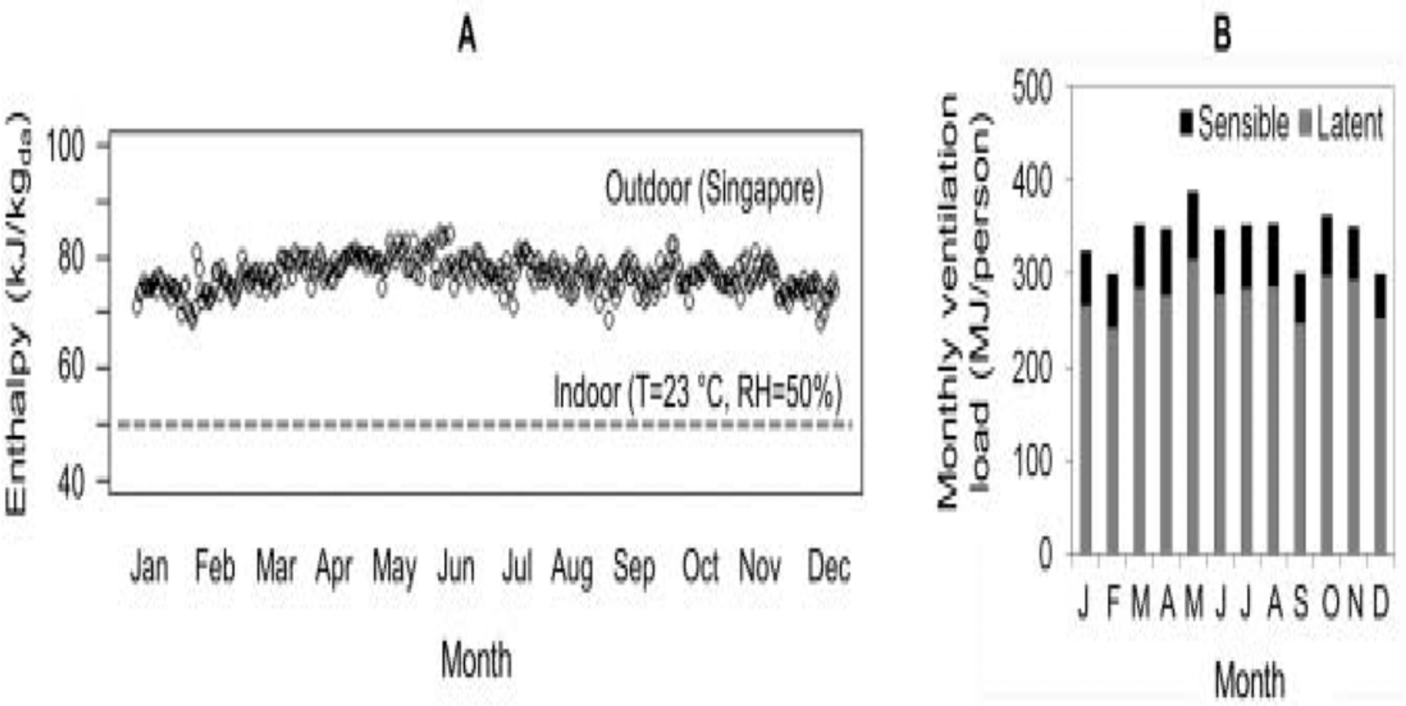

Fig 2. Enthalpy of outdoor and indoor air in Singapore. (A) Enthalpy of moist air for outdoor and indoor conditions. The plotted outdoor condition represents the daily average for building operating hours (M-F 7 am to 7 pm); the indoor condition corresponds to temperature = 23°C, RH = 50%. (B) Monthly sensible and latent energy needs to condition air at a ventilation rate of 10 L/s per person for building operation hours (M-F 7 am to 7 pm).

doi:10.1371/journal.pone.0122310.g002

different seasons (for example, 20°C in winter and 26°C in summer) in the light of simultaneously pursuing comfort and energy savings goals. Use of a constant ventilation rate during building operating hours, as we have done here, might lead to an overestimation of ventilation energy use, especially for the case of buildings with energy-saving ventilation strategies (e.g. demand-controlled ventilation or energy recovery ventilation). Consequently, the results of this study reveal the maximum reference energy cost for mechanically ventilated buildings in a specific tropical climate.

Results and Discussion

Results and discussion are presented in the following order: 1) ventilation energy demand; 2) ventilation energy cost in relation to the indoor set-point temperature and RH; 3) effects of COP and unit price of electricity cost on ventilation energy and cost; and 4) limitations and implications of the study.

Ventilation energy demand

Fig 2A summarizes the enthalpy profile of outdoor and indoor air. The outdoor air enthalpy is almost uniform throughout the year, with a mean value of 77 kJ/kg of dry air. In Singapore, the enthalpy of outdoor air is consistently higher than that of indoor air because of the warm and humid outdoor conditions. For an indoor condition of 23°C and 50% RH, the median (interquartile range) hourly difference between indoor and outdoor enthalpy for the whole year is 27 kJ/kg_{da} (23–30 kJ/kg_{da}). This enthalpy difference, combined with a ventilation rate of 10 L/s per person during the building operating hours (M-F 7 am to 7 pm), yields a total enthalpy-associated energy requirement of 4,592 MJ/y per person for buildings in Singapore's climate.

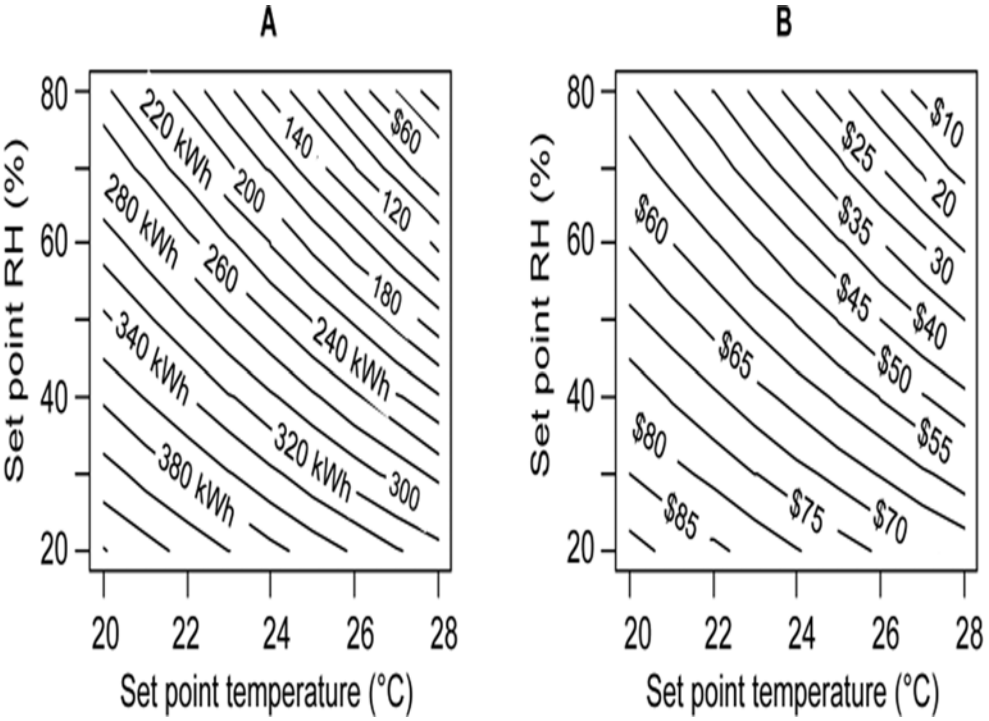

Fig 3. Baseline ventilation energy requirements and cost in Singapore. (A) Annual ventilation energy consumption (kWh/y per person) in relation to the indoor temperature and RH set point to provide 10 L/s per person with a COP = 4 during building operating hours, weekdays (M-F) from 7 am to 7 pm. (B) Annual electricity cost (US dollar ($)/y per person) for providing 10 L/s per person. (Analysis uses an electricity cost of 0.26 Singapore dollars per kWh, valid as of September 2014; Source: Singapore Power Tariff Rates [46]). Note: US$1 = S$1.25 as of September 2014.

doi:10.1371/journal.pone.0122310.g003

The energy required to treat ventilation air has two components: sensible to adjust temperature and latent to adjust moisture content (Fig 2B). The estimated annual loads associated with a building ventilation rate of 10 L/s per person in Singapore are 826 MJ/person for sensible heat and 3,766 MJ/person for latent heat, respectively. In a tropical climate, a large fraction—82% in this case—of the total ventilation energy load is associated with the requirement to remove latent heat from the air. This result highlights a key point: effective dehumidification of outdoor moist air is a critical component of building ventilation system performance in a tropical climate.

Ventilation energy cost in relation to the indoor set-point temperature and RH

Ventilation energy for treating outdoor air varies with the indoor thermal set points (temperature and RH). For a given indoor set point, the efficiency of air conditioning can be parameterized in terms of an "air conditioning system coefficient of performance" (COP). This term is defined as the ratio of the enthalpy change achieved in the air stream normalized by the electrical energy required to achieve it. The COP for compressor-based air conditioning systems might vary between 2 and 6 depending on temperature, RH, characteristics of the chiller, the cooling coil and the air distribution system [47]. Modern, high quality equipment can attain a COP higher than 4 [48, 49]. Considering a COP value of 4 and the building system operating hours (M-F 7 am to 7 pm), Fig 3 presents the annual ventilation energy consumption (kWh/y per person) and associated cost in relation to the set point of indoor temperature and RH. The

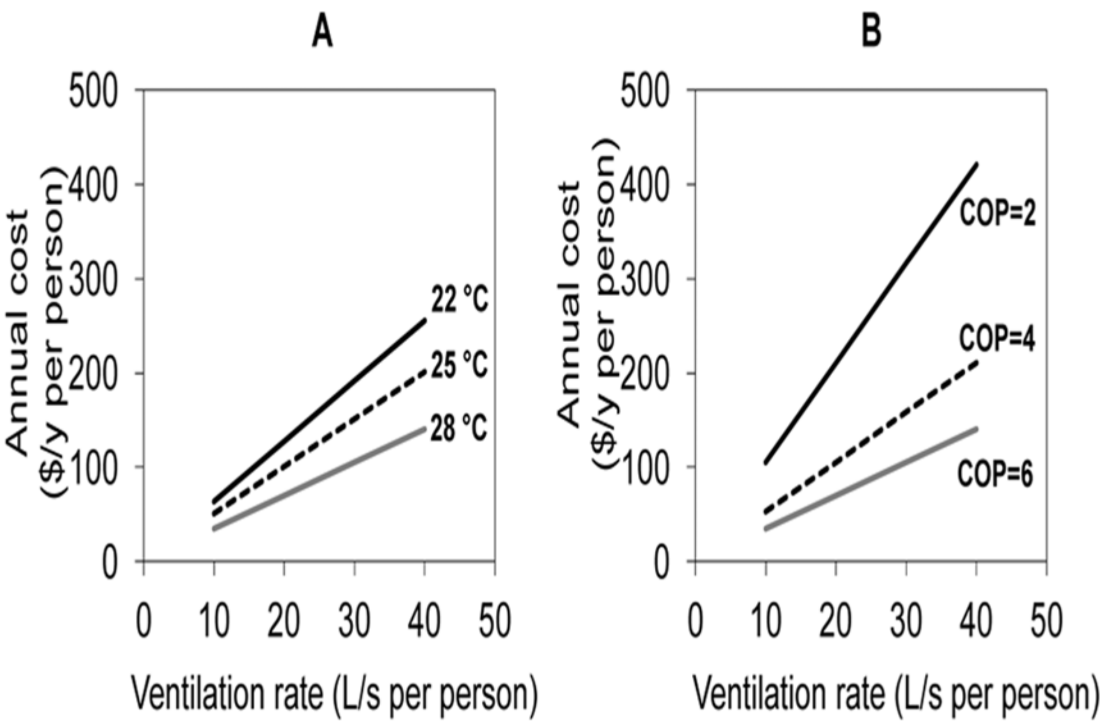

Fig 4. Ventilation energy requirements and cost in Singapore in relation to ventilation rate. (A) Annual cost (US$) and energy use with thermal conditioning of ventilation air for varied indoor set-point temperature (with COP = 4 and RH = 50%). (B) Annual cost and energy use associated with thermal conditioning of ventilation air for different COP values (temperature = 25°C and RH = 50%). Electricity cost assumed to be US$0.21 (= S$0.26) per kWh. Calculations are based on operating the ventilation system from 7 am to 7 pm on all weekdays (M-F) of the year.

doi:10.1371/journal.pone.0122310.g004

contour lines are labeled with the total annual energy consumption (Fig 3A) and the associated total annual cost (Fig 3B) for providing a ventilation flow rate of 10 L/s per person.

The figures illustrate the extent to which ventilation energy and cost decrease with increased indoor set points for temperature and RH. Under indoor conditions of moderate temperature (> 26°C) and RH, an increase in ventilation airflow rate can be achieved at relatively low cost. For example, the energy cost for conditioning 10 L/s per person of ventilation air to a temperature of 26°C and an RH of 60% can be accomplished in the Singapore climate at an annual cost of only US $40/y per person. For lower temperature (22°C) and RH (40%) conditions, the cost of providing 10 L/s per person is increased to approximately US $70/y.

Fig 4A shows the annual ventilation cost (left *y*-axis) for increasing ventilation rate as a function of indoor set-point temperature (22°C, 25°C, and 28°C), assuming an indoor RH of 50% and a COP value of 4. Studies indicate that ventilation rates less than 10 L/s per person are associated with an increased prevalence of sick building syndrome symptoms, whereas rates higher than 25 L/s per person can significantly reduce building-related health symptoms [1, 2, 7, 39]. According to Fig 4A, the ventilation cost for providing a volume flow rate of 10 L/s per person (with COP of 4) is less than US $ 70/y per person for all indoor set-point temperatures considered. The per-person cost varies between $ 85/y (for 28°C) and $ 160/y (for 22°C) for achieving 25 L/s per person. Such costs are low in relation to annual wages of office workers in Singapore. Enhanced ventilation flow rates are well justified economically if they yield even small improvements in workplace productivity. Furthermore, it appears possible to achieve thermal comfort in a tropical climate with indoor temperatures that are higher than the normal

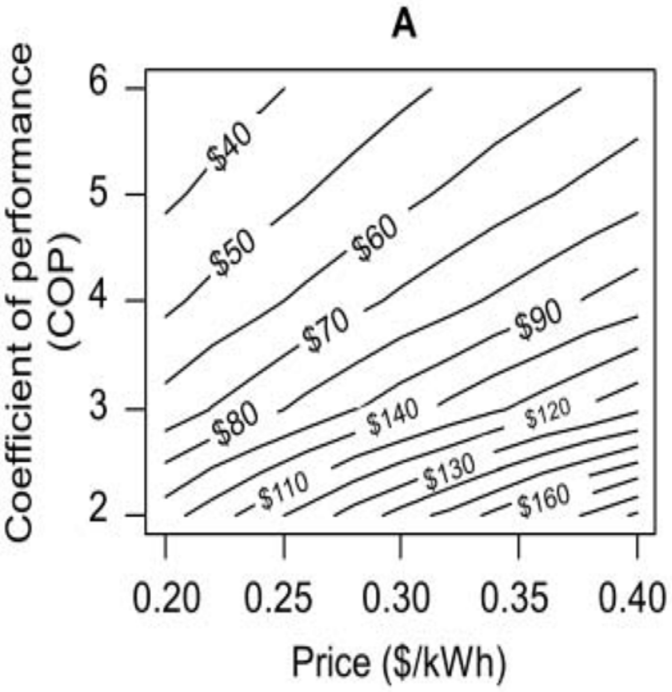

Fig 5. Cost of conditioning ventilation air in Singapore in relation to air conditioning system performance and electricity price. Effect of COP and the unit cost of electricity on ventilation energy cost (US$/y per person) assuming a set point temperature = 25°C and RH = 50%. (A) Ventilation rate = 10 L/s per person and (B) Ventilation rate = 25 L/s per person. The analysis is based on an office-building operating schedule (7 am to 7 pm all weekdays of the year, Monday-Friday).

doi:10.1371/journal.pone.0122310.g005

set point for air conditioning in temperate zones [41–45]. If so, then even a large increase in the ventilation rate could be realized with a small net energy cost for thermal conditioning.

The efficiency of the air conditioning system, as parameterized by the COP, has an important influence on the outcomes assessed here. Fig 4B shows the annual ventilation cost for providing a specified ventilation rate as a function of the overall system COP, within the range of 2 to 6. As the figure illustrates, the annual ventilation cost decreases as COP increases. The per-person ventilation cost for providing 25 L/s per person is up to $ 265/y if the COP is 2, whereas the cost is less than $ 90/y with a COP of 6. For a given system, COP depends on the air conditioning cycle, refrigerant, and system operating temperature and humidity. A high performance air conditioning system (high COP) can be influential in saving energy. Comparing Fig 4A and 4B illustrates that varying the COP across the range 2 to 4 has a much more significant impact on energy cost than varying the indoor set-point temperature across the range of 22°C to 28°C.

Effects of COP and unit price of electricity on ventilation energy and cost

Owing to advances in technology, building mechanical system performance is expected to improve with time. However, with increasing population and energy demand, the cost of electricity is also expected to increase during the coming decades. Fig 5 depicts the ventilation energy cost as a function of the overall system COP and the unit cost of electricity. In the two frames, the x-axis and the y-axis represent the unit cost of electricity and the air conditioning system COP, respectively. The contour lines are labeled with the total annual cost (US$/y per person) for providing ventilation flow rates of 10 L/s per person (Fig 5A) and 25 L/s per person

(Fig 5B). The annual cost for thermal conditioning ventilation air is proportional to the unit cost of electricity. Improved system performance can compensate for increasing energy costs. For instance, for a unit electricity cost of 0.40 US$/kWh (~ 90% higher than the price as of 5 September 2014 in Singapore), providing a ventilation rate of 10 L/s per person would cost $226/y per person with a COP of 2, whereas, with a COP value of 5, thermally conditioning the same ventilation rate can be achieved at an annual cost of less than $90/y per person (Fig 5A). Even at significantly increased cost of electricity such as 0.40 US$/kWh, a system with a high COP (> 4) can sustain thermal conditioning of ventilation at a rate of 25 L/s per person at an annual cost of less than $285 (Fig 5B).

Assessing the benefits of an elevated ventilation rate

Multidisciplinary reviews of the scientific literature suggest that providing a building ventilation rate of 25 L/s per person can enhance the productivity and health of office workers; associated health-related benefits exceed the associated energy costs [1, 7]. Fisk et al. [2] reported that as the ventilation rate decreases from 10 to 5 L/s per person, relative sick building syndrome (SBS) symptom prevalence increases approximately 23% (confidence interval: 12% to 32%), and as the rate increases from 10 to 25 L/s per person, relative prevalence decreases approximately 29% (15% to 42%). A later study by Fisk et al. [50] evaluated changes in sick building syndrome (SBS) symptoms, work performance, and short-term absence for four ventilation scenarios of office buildings. The total economic benefit was estimated to be $5.6 billion per year for increasing ventilation rates to 10 L/s per person in US office buildings, and $13.5 billion for increasing ventilation rates to 15 L/s per person, considering decreased SBS prevalence, reduced short-term leaves, and productivity increases. Based on experimental observations, Wargocki et al. [8] reported that performance of office-type tasks (text typing, proof-reading, addition) improved monotonically with increasing ventilation rates. On average, for each doubling of ventilation rate in the range between 3 and 30 L/s per person, office task performance improved by an average of 1.7%. Fisk and Seppänen [51] reported benefit-cost ratios as high as 80 and net economic benefits as high as $700 per person per year attributable to ventilation rate increases in offices and schools in the US.

The present study indicates that in a tropical climate, such as Singapore's, ventilation rates as high as 25 L/s per person can be achieved at a moderate cost (< US $300/y per person). This cost is less than 1% of the median gross income of an office worker in Singapore, approximately US$34,000/y per person (corresponding to S$42,000/y per person) as of June 2013 [52]. Hence, even a 1% improvement in productivity that results from providing high ventilation rates would be economically justified. A study performed in Singapore [53] showed significantly better performance (by 22%) as measured by average talk time with a higher ventilation rate (10 L/s per person) than with a lower ventilation rate (5 L/s per person) when the temperature was 24.5°C. The study found a small (and not statistically significant) reduction in performance with a higher ventilation rate when the temperature was 22.5°C. The results of this study hint that the increased ventilation rate has larger impacts on improving productivity when the indoor set-point temperature is higher than 24.5°C. Seppänen et al. [54] reported up to 3% improvement in average performance per 10 L/s per person increase in outdoor air ventilation rate. That study also indicated that the performance increase per unit increase in ventilation was more significant at lower ventilation rates (< 20 L/s per person) and nearly negligible at rates over 45 L/s person.

Even though reducing building energy consumption in tropical climates is an important goal because of the energy required for conditioning outdoor air, the energy cost attributable to establishing and maintaining high ventilation rates is much lower (<1%) than annual wage of

office workers. Even in Singapore's tropical climate, building occupants can be provided with substantial ventilation airflow rates in a cost effective way, thereby minimizing exposure to contaminants of indoor origin and promoting occupant health and comfort of occupants at only modest net cost.

Study limitations and implications

This paper has presented the results of an analysis to assess energy use and costs due to increased ventilation rates for office buildings in a tropical climate. Evidence from several published studies supports the benefits of providing higher than customary outdoor air ventilation rates. The results of the present study suggests that the cost of thermal conditioning even a high flow rate of ventilation air is very much less than the annual salary of the average office worker in Singapore, regardless of indoor thermal set point conditions. Specifically, a case study conducted in Singapore [53] has suggested that increased ventilation rate can have substantial impacts on productivity especially when the indoor set-point temperature is higher than 24.5 C. Furthermore, it seems that in tropical climates thermal comfort can be achieved with indoor temperatures higher than the normal set point for air conditioning in temperate zones if alternative or complementary cooling measures are provided, especially if these supplementary measures are under occupant control (e.g., ceiling or desk fans) [41–45]. This study has focused on the energy required to provide specified ventilation rates for the climate conditions in Singapore. Energy required to bring the outdoor air to the set-point conditions was estimated to obtain results that are independent of specific building features or mechanical system characteristics. The relationship between ventilation rate per person and associated energy use for conditioning ventilation air is theoretically linear based on the energy conservation principle. Building system factors such as fan efficiency, pressure losses due to duct geometry, size, length, and junctions, energy losses associated with air handling units (i.e., filters, heat exchangers) contribute to deviations from the linear relationship. These factors vary with the building size, building type, and HVAC system and operation characteristics. The impacts of specific building characteristics and occupant behaviors on the energy use are not within the scope of the present study. Nonetheless, the approach used in this study provides meaningful insight into ventilation-specific energy requirements for office buildings in tropical climates. The results obtained are general for the given climate, independent of specific building characteristics. The energy load for dehumidification and cooling of mechanical ventilation for office buildings is fairly consistent throughout the year. This trend is mainly a result of consistent requirement for dehumidification in a tropical climate and constant indoor set-point conditions in office buildings, despite changes in daily rainfall in different seasons.

The present study provides fundamental insights into the energy cost for thermal conditioning ventilation air in a tropical climate. However, it should be noted that there are several ways to cool and dehumidify the air (e.g., cooling with condensation, cooling without condensation, using liquid or solid desiccant, etc.). A follow-up study with particular attention to variation in type of cooling could address how the sensible and latent cooling processes can vary in practice for office buildings in tropical climate. Furthermore, applying energy-saving technologies such as energy recovery ventilators (ERV) would reduce the energy consumption attributable to ventilation. In addition, concepts such as demand controlled ventilation, displacement ventilation, underfloor air distribution, and personal environmental control systems [55–57], have potential to reduce further the gross demand for ventilation by improving the effectiveness of utilizing ventilation air.

Conclusion

We have estimated energy and cost for thermal conditioning of ventilation air for a tropical climate. The energy consumption rate (kWh/y per person) and associated cost were calculated as a function of ventilation rate (L/s per person), indoor set-point temperature and relative humidity, overall system performance, and the cost of electricity. The results show that in a tropical climate, effective dehumidification of outdoor moist air is a critical aspect of building ventilation system performance. For specific indoor climate conditions, the energy cost for ventilation varies with system performance (as measured by the COP) and the unit cost of electricity. Overall, even in a thermally demanding tropical climate, the energy cost associated with increasing ventilation rate up to 25 L/s per person is less than 1% of the wages of an office worker in an advanced economy like Singapore's. Improvements in workplace performance would exceed the costs of providing high ventilation flow rates in office buildings especially when the indoor set-point temperature is higher than 24°C. Using appropriate strategies (e.g., augmented convective cooling with fans) with higher than typical indoor set-point temperature (> 26°C) can allow for a high ventilation rate (such as 25 L/s per person) at a relatively modest thermal conditioning cost.

Author Contributions

Conceived and designed the experiments: DR SS WWN. Performed the experiments: DR. Analyzed the data: DR SS WWN. Contributed reagents/materials/analysis tools: DR SS WWN. Wrote the paper: DR SS WWN.

References

1. Sundell J, Levin H, Nazaroff WW, Cain WS, Fisk WJ, Grimsrud DT, et al. (2011) Ventilation rates and health: multidisciplinary review of the scientific literature. Indoor Air 21: 191–204. doi: 10.1111/j.1600-0668.2010.00703.x PMID: 21204989
2. Fisk WJ, Mirer AG, Mendell MJ (2009) Quantitative relationship of sick building syndrome symptoms with ventilation rates. Indoor Air 19: 159–165. doi: 10.1111/j.1600-0668.2008.00575.x PMID: 19207289
3. Frontczak M, Schiavon S, Goins J, Arens E, Zhang H, Wargocki P (2012) Quantitative relationships between occupant satisfaction and satisfaction aspects of indoor environmental quality and building design. Indoor Air 22: 119–131. doi: 10.1111/j.1600-0668.2011.00745.x PMID: 21929547
4. Mendell MJ, Lei-Gomez Q, Mirer AG, Seppänen OA, Brunner G (2008) Risk factors in heating, ventilating, and air-conditioning systems for occupant symptoms in US office buildings: the US EPA BASE study. Indoor Air 18: 301–316. doi: 10.1111/j.1600-0668.2008.00531.x PMID: 18492050
5. Bernstein JA, Alexis N, Bacchus H, Bernstein IL, Fritz P, Horner E, et al. (2008) The health effects of nonindustrial indoor air pollution. J Allergy Clin Immunol 121: 585–591. PMID: 18155285
6. Li Y, Leung GM, Tang JW, Yang X, Chao CYH, Lin JZ, et al. (2007) Role of ventilation in airborne transmission of infectious agents in the built environment—a multidisciplinary systematic review. Indoor Air 17: 2–18. PMID: 17257148
7. Wargocki P, Sundell J, Bischof W, Brundrett G, Fanger PO, Gyntelberg F, et al. (2002) Ventilation and health in non-industrial indoor environments: report from a European Multidisciplinary Scientific Consensus Meeting (EUROVEN). Indoor Air 12: 113–128. PMID: 12216467
8. Wargocki P, Wyon DP, Sundell J, Clausen G, Fanger PO (2000) The effects of outdoor air supply rate in an office on perceived air quality, sick building syndrome (SBS) symptoms and productivity. Indoor Air 10: 222–236. PMID: 11089327
9. Menzies D, Bourbeau J (1997) Building-related illnesses. N Engl J Med 337: 1524–1531. PMID: 9366585
10. Hodgson M (2002) Indoor environmental exposures and symptoms. Environ Health Perspect 110 (Suppl 4): 663–667. PMID: 12194903
11. Wolkoff P, Wilkins CK, Clausen PA, Nielsen GD (2006) Organic compounds in office environments—sensory irritation, odor, measurements and the role of reactive chemistry. Indoor Air 16: 7–19. PMID: 16420493

12. Mundt E, Mathisen HM, Nielsen PV, Moser A (2004) Ventilation effectiveness. REHVA Guidebook No. 2.
13. Rim D, Novoselac A (2008) Transient simulation of airflow and pollutant dispersion under mixing flow and buoyancy driven flow regimes in residential buildings. ASHRAE Transactions 114: 130–142.
14. Melikov AK (2004) Personalized ventilation. Indoor Air 14 (Suppl 7): 157–167. PMID: 15330783
15. Singapore Standard (2009) Code of practice-air conditioning and mechanical ventilation in buildings (SS553), SPRING Singapore.
16. ENV Ministry of the Environment (1996) Singapore guidelines for good indoor air quality in office premises.
17. ANSI/ASHRAE (2013) Ventilation for acceptable indoor air quality. American Society of Heating. Standard 62.1. Refrigerating and Air-Conditioning Engineers.
18. Olesen BW (2004) International standards for the indoor environment. Indoor Air 14 (Suppl 7): 18–26. PMID: 15330767
19. Harrison PTC (2002) Indoor air quality guidelines. Occup Environ Med 59: 73–74. PMID: 11850548
20. CEN (2007) Criteria for the indoor environment including thermal, indoor air quality, light and noise. EN 15251–2007. European Committee for Standardization.
21. Bennett DH, Fisk W, Apte MG, Wu X, Trout A, Faulkner D, et al. (2012) Ventilation, temperature, and HVAC characteristics in small and medium commercial buildings in California. Indoor Air 22: 309–320. doi: 10.1111/j.1600-0668.2012.00767.x PMID: 22257121
22. Persily A, Gorfain J (2008) Analysis of ventilation data from the U.S. Environmental Protection Agency Building Assessment Survey and Evaluation (BASE) study, NISTIR 7145-Revised. Washington, DC: National Institute of Standards & Technology.
23. Cummings JB, Withers CR, Moyer N, Fairey P, McKendry B (1996) Uncontrolled air flow in non-residential buildings. Florida Solar Energy Center.
24. USDOE (2011) Report on the First Quadrennial Technology Review. U.S. Department of Energy.
25. Pérez-Lombard L, Ortiz J, Pout C (2008) A review on buildings energy consumption information. Energy Build 40: 394–398.
26. Lam JC, Wan KKW, Yang L (2008) Sensitivity analysis and energy conservation measures implications. Energ Convers Manage 49: 3170–3177.
27. Fridley DG, Zheng N, Zhou N (2008) Estimating total energy consumption and emissions of China's commercial and office buildings. Berkeley, CA: Lawrence Berkeley National Laboratory Report No. LBNL-248E.
28. USEPA (2009) Buildings and their impact on the environment: a statistical summary. U.S. Environmental Protection Agency.
29. Singapore NEA (2008) Annual Report. Singapore National Environment Agency.
30. Saidur R (2009) Energy consumption, energy savings, and emission analysis in Malaysian office buildings. Energy Policy 37: 4104–4113.
31. Lowe R (2007) Addressing the challenges of climate change for the built environment. Build Res Inf 35: 343–350.
32. Frank TH (2005) Climate change impacts on building heating and cooling energy demand in Switzerland. Energy Build 37: 1175–1185.
33. Belzer DB, Scott MJ, Sands RD (1996) Climate change impacts on U.S. commercial building energy consumption: an analysis using sample survey data. Energy Sources 18: 177–201.
34. Iqbal I, Al-Homoud MS (2007) Parametric analysis of alternative energy conservation measures in an office building in hot and humid climate. Build Environ 42: 2166–2177.
35. Nazaroff WW (2013) Exploring the consequences of climate change for indoor air quality. Environmental Research Letters 8: 015022.
36. Lee SE, Rajagopalan P (2008) Building energy efficiency labeling programme in Singapore. Energy Policy 36: 3982–3992.
37. ASHRAE (2002) International weather for energy calculations (IWEC Weather Files) User's Manual, Version 1.1. American Society of Heating, Refrigerating and Air-Conditioning Engineers.
38. ASHRAE (2013) ASHRAE Handbook-Fundamentals. American Society of Heating, Refrigerating and Air-Conditioning Engineers.
39. Fisk WJ, Black D, Brunner G (2012) Changing ventilation rates in U.S. offices: Implications for health, work performance, energy, and associated economics. Build Environ 47: 368–372.
40. Hinds WC (1999) Aerosol technology: properties, behavior, and measurement of airborne particles. 2nd ed. John Wiley & Sons.

41. Zhang H, Arens E, Kim D, Buchberger E, Bauman F, Huizenga C (2010) Comfort, perceived air quality, and work performance in a low-power task-ambient conditioning system. Build Environ 45: 29–39.
42. Cândido C, de Dear RJ, Lamberts R, Bittencourt L (2010) Air movement acceptability limits and thermal comfort in Brazil's hot humid climate zone. Build Environ 45: 222–229. doi: 10.1080/03601231003613609 PMID: 20390954
43. Schiavon S, Melikov AK (2008) Energy saving and improved comfort by increased air movement. Energy Build 40: 1954–1960.
44. Hwang RL, Cheng MJ, Lin TP, Ho MC (2009) Thermal perceptions, general adaptation methods and occupant's idea about the trade-off between thermal comfort and energy saving in hot–humid regions. Build Environ 44: 1128–1134.
45. Nicol F (2004) Adaptive thermal comfort standards in the hot–humid tropics. Energy Build 36: 628–637.
46. EMA Singapore (2014) Singapore Power Tariff Rates, Energy Market Authority. Available: http://www.singaporepower.com.sg. Accessed 2014 Sep 5.
47. Adnot J, Greslou O, Riviere P, Spadaro J, Hitchin R, Pout C, et al. (2012) Final report of task 2—air-conditioning products, Sustainable industrial policy—building on the ecodesign directive—energy-using product. European Commission.
48. Lam JC (2000) Energy analysis of commercial buildings in subtropical climates. Build Environ 35: 19–26.
49. Sekhar SC (2005) Space temperature difference, cooling coil and fan—energy and indoor air quality issues revisited. Energy Build 37: 49–54.
50. Fisk WJ, Black D, Brunner G (2011) Benefits and costs of improved IEQ in U.S. offices. Indoor Air 21: 357–367. doi: 10.1111/j.1600-0668.2011.00719.x PMID: 21470313
51. Fisk WJ, Seppänen OA (2007) Providing better indoor environment quality brings economic benefits. Proceedings of the Clima 2007 Wellbeing Indoors Conference.
52. Republic of Singapore (2013) Income: Gross Monthly Income from Work. Singapore Ministry of Manpower.
53. Tham KW (2004) Effects of temperature and outdoor air supply rate on the performance of call center operators in the tropics. Indoor Air 14 (Suppl 7): 119–125. PMID: 15330779
54. Seppänen OA, Fisk WJ, Lei QH (2006) Ventilation and performance in office work. Indoor Air 16: 28–36. PMID: 16420495
55. Persily A, Emmerich SJ (2012) Indoor air quality in sustainable, energy efficient buildings. HVAC&R Research 18: 4–20.
56. Sekhar SC, Gong N, Tham KW, Cheong KW, Melikov AK, Wyon DP, et al. (2005) Findings of personalized ventilation studies in a hot and humid climate. HVAC&R Research 11: 603–620.
57. Schiavon S, Melikov AK, Sekhar C (2010) Energy analysis of the personalized ventilation system in hot and humid climates. Energy Build 42: 699–707.
